# Supplementary figures and images for: PACSIN1 is indispensable for amphisome-lysosome fusion during basal autophagy and subsets of selective autophagy
Source: PLoS Genet. 2022 Jun 30;18(6):e1010264. doi: 10.1371/journal.pgen.1010264 (PMC9246181; doi:10.1371/journal.pgen.1010264)

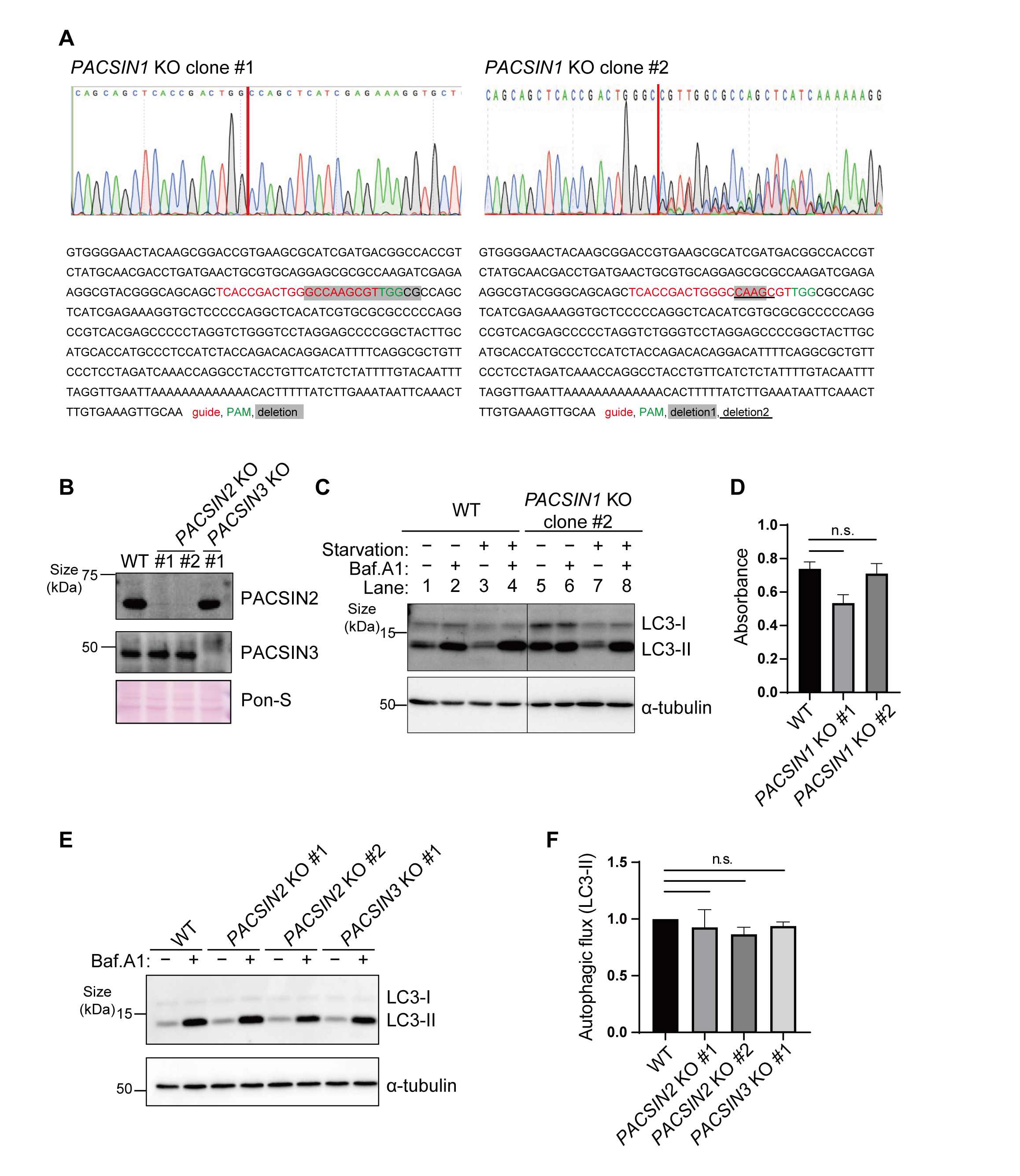

Supplement: S1 Fig — (A) Isolated PACSIN1 KO clone #1 HeLa cells exhibited a 14-base deletion and isolated PACSIN1 KO clone #2 HeLa cells exhibited hemizygous deletion at the indicated locus on the second exon of PACSIN1. The PAM sequence and recognition sequence are labeled in green and red, respectively. (B) Immunoblot confirming the absence of protein in established PACSIN2 and PACSIN3 KO HeLa cells. (C) WT or PACSIN1 KO clone #2 HeLa cells were cultured for 2 h in growth medium (DMEM, NT) or starvation medium (EBSS, ST) with 125 nM Baf.A1 and then analyzed by immunoblot using anti-LC3 and anti–α-tubulin antibodies. PACSIN1 KO clone #2 HeLa cells exhibited impairment of autophagic activity similar with PACSIN1 KO clone #1 HeLa cells. (D) Cell proliferation assay performed by WST-8 after 24 h seeded WT and PACSIN1 KO clone #1, #2 HeLa cells, mean ± s.e.m (n = 3). n.s.; not significant (one-way ANOVA with Dunnett’s multiple comparisons test). (E) WT, PACSIN2, and PACSIN3 KO HeLa cells were cultured for 2 h in growth medium with 125 nM Baf.A1 and then analyzed by immunoblotting using anti-LC3 and anti–α-tubulin antibodies. (F) LC3 flux was quantified, mean ± s.e.m (n = 3). n.s.; not significant (one-way ANOVA with Dunnett’s multiple comparisons test). (TIF) [file pgen.1010264.s001.tif]

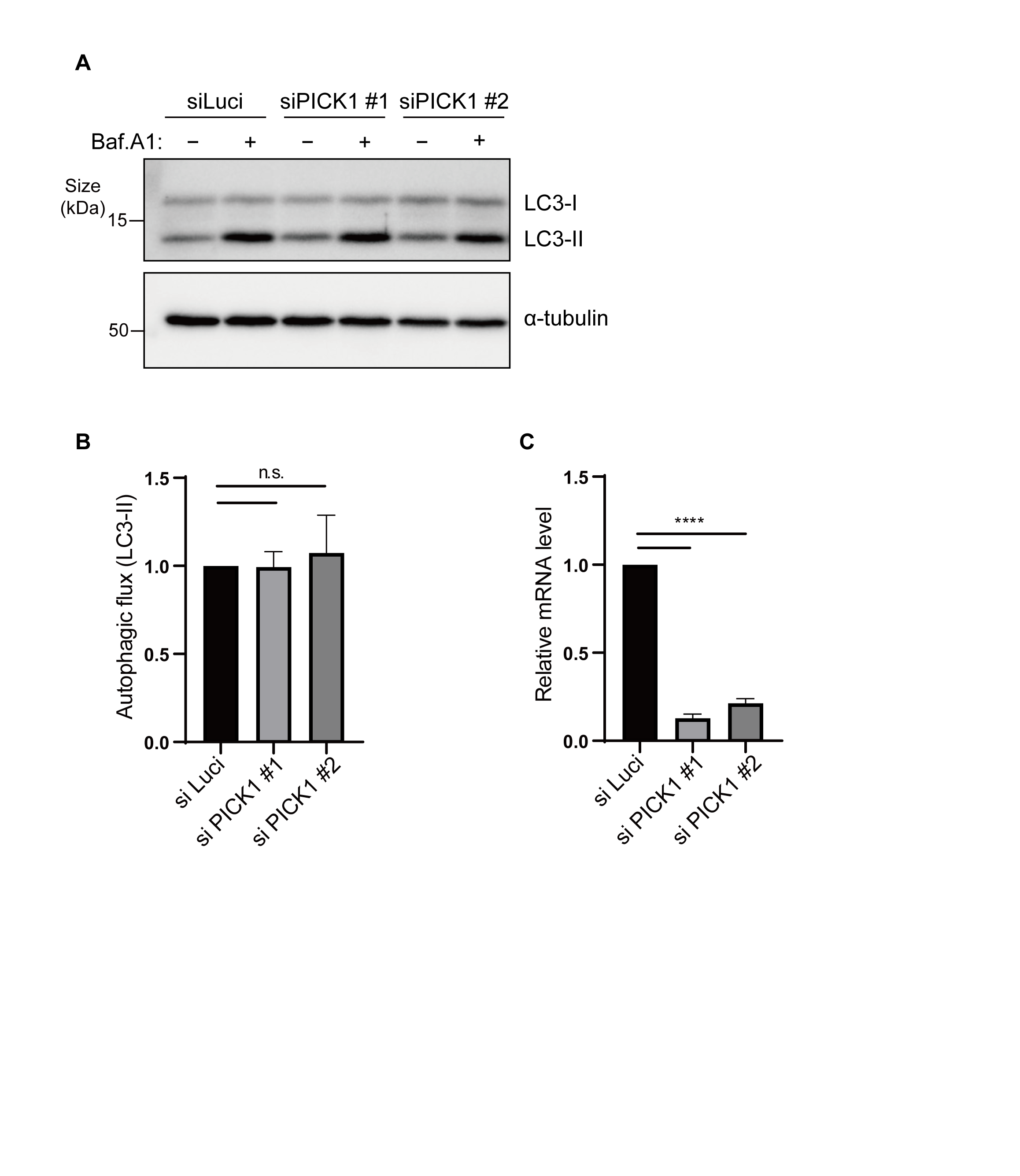

Supplement: S2 Fig — (A) WT or PACSIN1 KO HeLa cells treated with siLuciferase or siPICK1 were cultured for 2 h in growth medium with or without 125 nM Baf.A1, then analyzed by immunoblot using anti-LC3 and anti–α-tubulin antibodies. (B) LC3 flux was quantified, mean ± s.e.m (n = 4). n.s.; not significant (one-way ANOVA with Dunnett’s multiple comparisons test). (C) Gene expression of PICK1 was quantified by real-time PCR (qRT-PCR), mean ± s.e.m (n = 4). P value (****p < 0.0001) was determined by one-way ANOVA with Dunnett’s multiple comparisons test. (TIF) [file pgen.1010264.s002.tif]

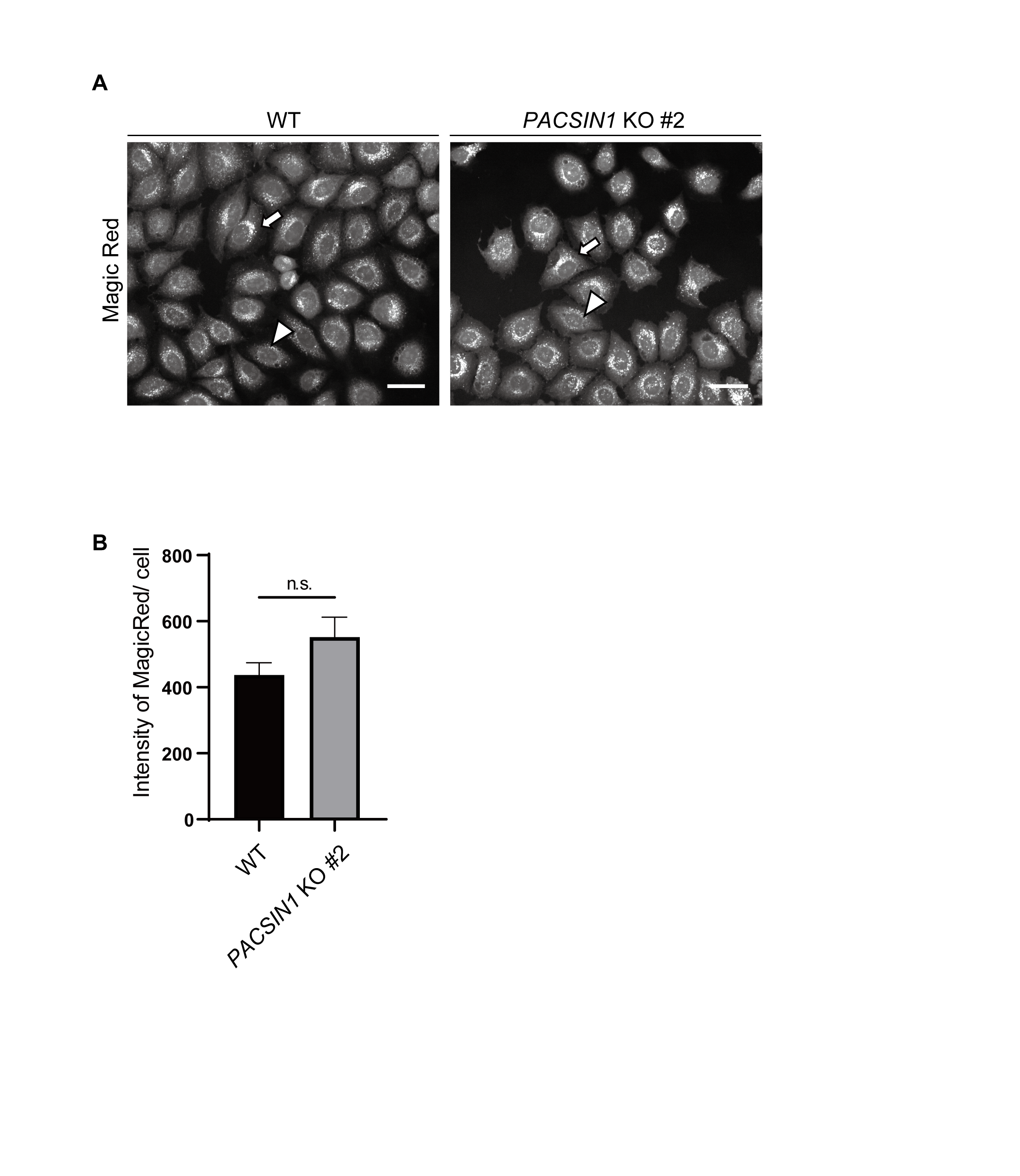

Supplement: S3 Fig — (A) WT or PACSIN1 KO clone #2 HeLa cells were cultured in growth medium and treated with Magic Red. After fixation, cells were analyzed using CQ1 software. The cell exhibited relatively high and low intensity of Magic Red were indicated by arrows and arrowheads, respectively. Scale bars, 40 μm. (B) Quantified Magic Red mean intensity normalized per cell, mean ± s.e.m. More than 200 cells were analyzed per condition in each experiment (n = 3). n.s.; not significant (the two-tailed, unpaired t-test). (TIF) [file pgen.1010264.s003.tif]

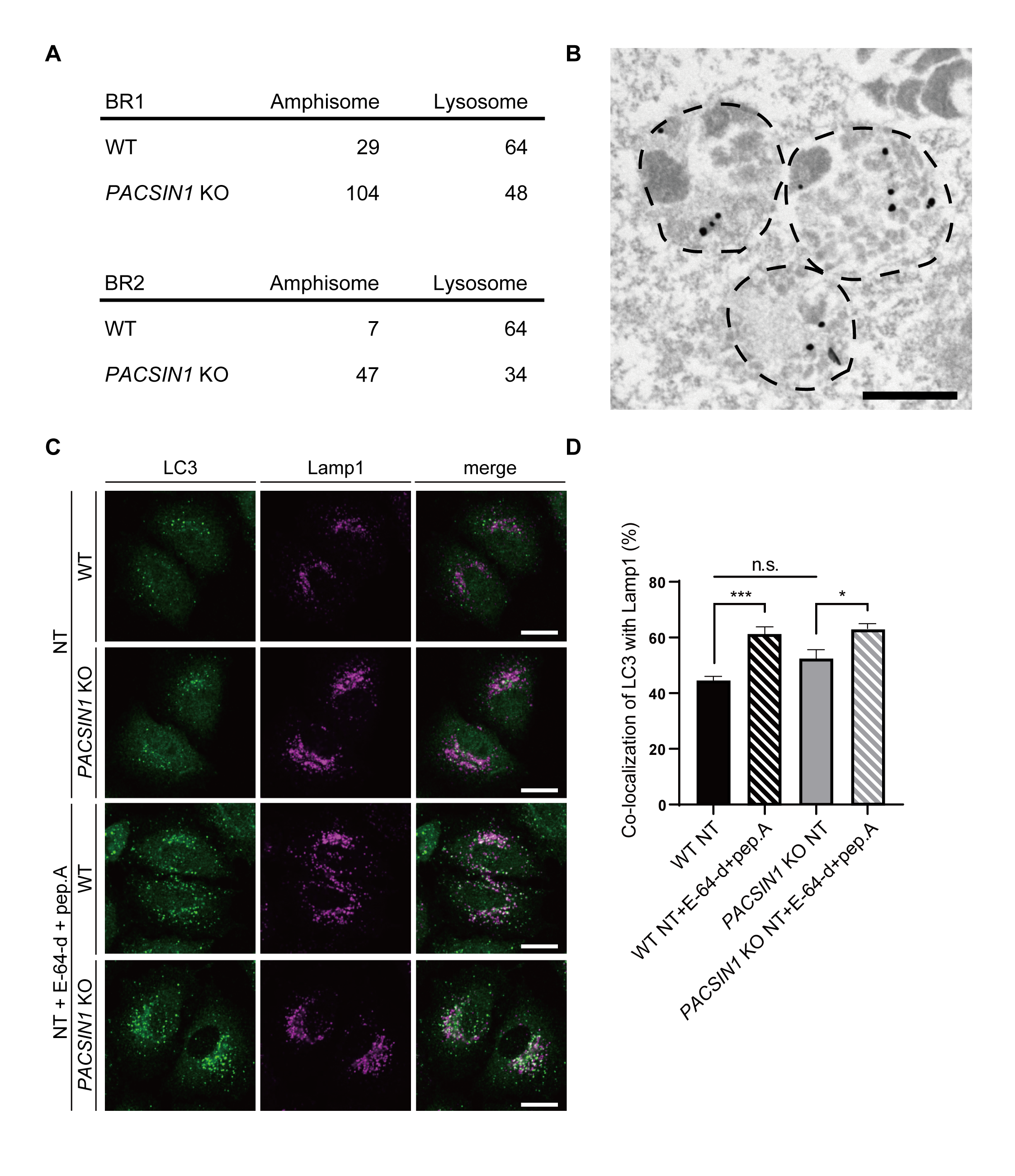

Supplement: S4 Fig — (A) Quantified the number of amphisome and lysosome in WT and PACSIN1 KO HeLa cells from total 80 images of two independent experiments. (B) Immunogold particles identifying LC3B are localized in vacuoles containing small vesicles in PACSIN1 KO HeLa cells. Scale bars, 500 nm. (C) WT or PACSIN1 KO HeLa cells stably expressing Lamp1-mcherry were cultured for 2 h in growth medium with or without 10 μg/mL E-64-d and pepstatin A. After fixation, cells were immunostained with anti-LC3 antibodies. Scale bars, 20 μm. (D) The co-localization rate of LC3 with Lamp1 was quantified using CQ1 software, mean ± s.e.m. More than 200 cells were analyzed per condition in each experiment (n = 5). n.s.; not significant, *p < 0.05, ***p < 0.001 (one-way ANOVA with Tukey’s multiple comparisons test). (TIF) [file pgen.1010264.s004.tif]

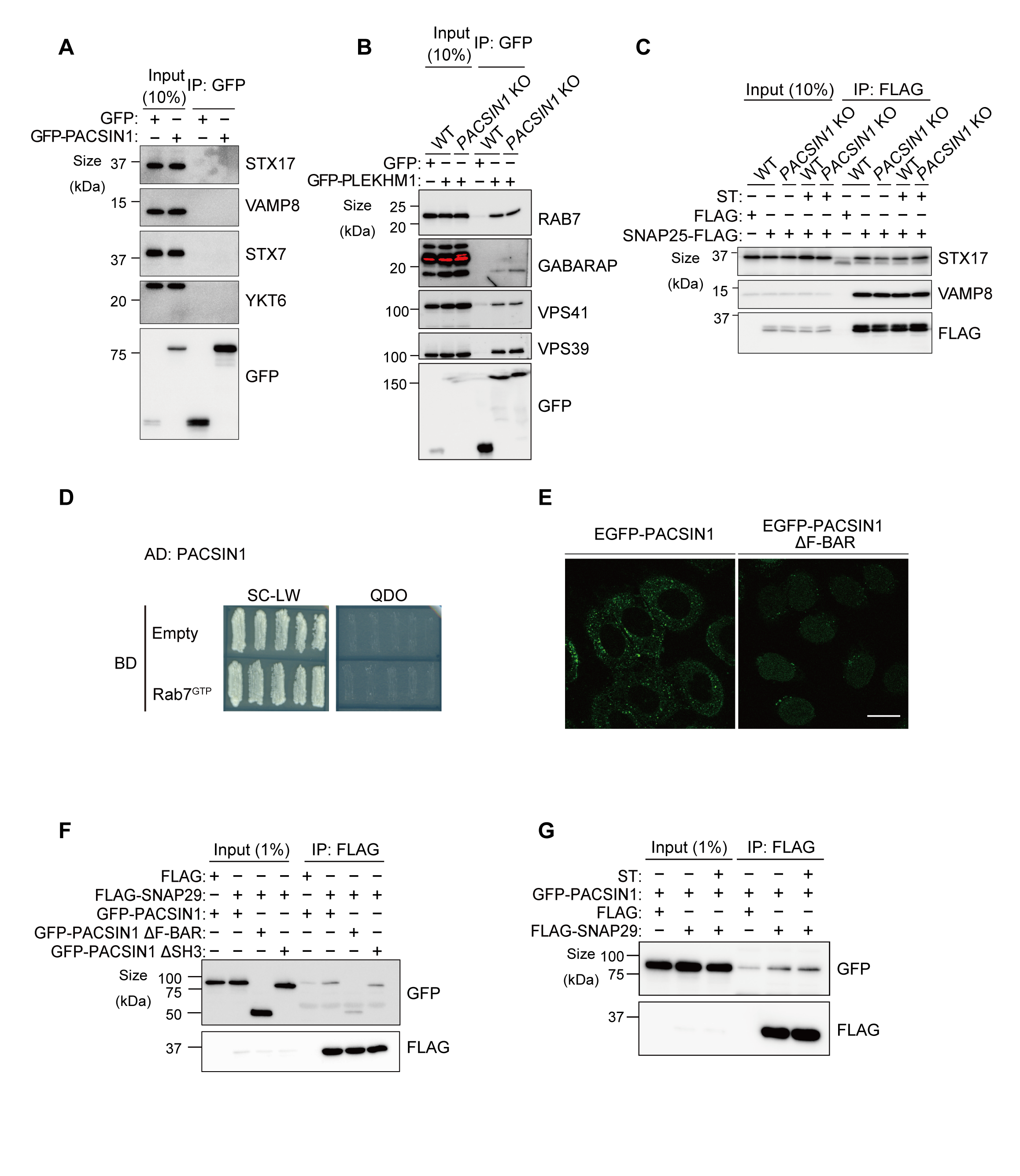

Supplement: S5 Fig — (A) WT HeLa cells were transfected with GFP-PACSIN1. The lysates were immunoprecipitated with GFP-trap beads and immunoblotted with the indicated antibodies. (B) WT and PACSIN1 KO HeLa cells were transfected with GFP-PLEKHM1. The lysates were immunoprecipitated with GFP-trap beads and immunoblotted with the indicated antibodies. (C) WT or PACSIN1 KO HeLa cells transiently expressing SNAP25-FLAG were cultured in growth medium or starvation medium (EBSS, ST) for 2 h. The lysates were immunoprecipitated with FLAG-M2 beads and immunoblotted with anti-VAMP8 and anti-STX17 antibody. (D) A Y2H assay showed that PACSIN1 did not interact with Rab7GTP. (E) WT HeLa cells stably expressing EGFP-PACSIN1 or EGFP-PACSIN1 ΔF-BAR. Cells were cultured for 2 h in growth medium with 125 nM Baf.A1. Cells were pre-treated with 0.05% saponin and fixed. The samples were analyzed by confocal microscopy. Scale bars, 20 μm. (F) WT HeLa cells were transfected with indicated plasmids. The lysates were immunoprecipitated with FLAG-M2 beads and immunoblotted with the indicated antibodies. (G) WT HeLa cells transiently expressing FLAG-SNAP29 and GFP-PACSIN1 were cultured in growth medium or starvation medium (EBSS, ST) for 2 h. The lysates were immunoprecipitated with FLAG-M2 beads and immunoblotted with anti-GFP and anti-FLAG antibody. (TIF) [file pgen.1010264.s005.tif]

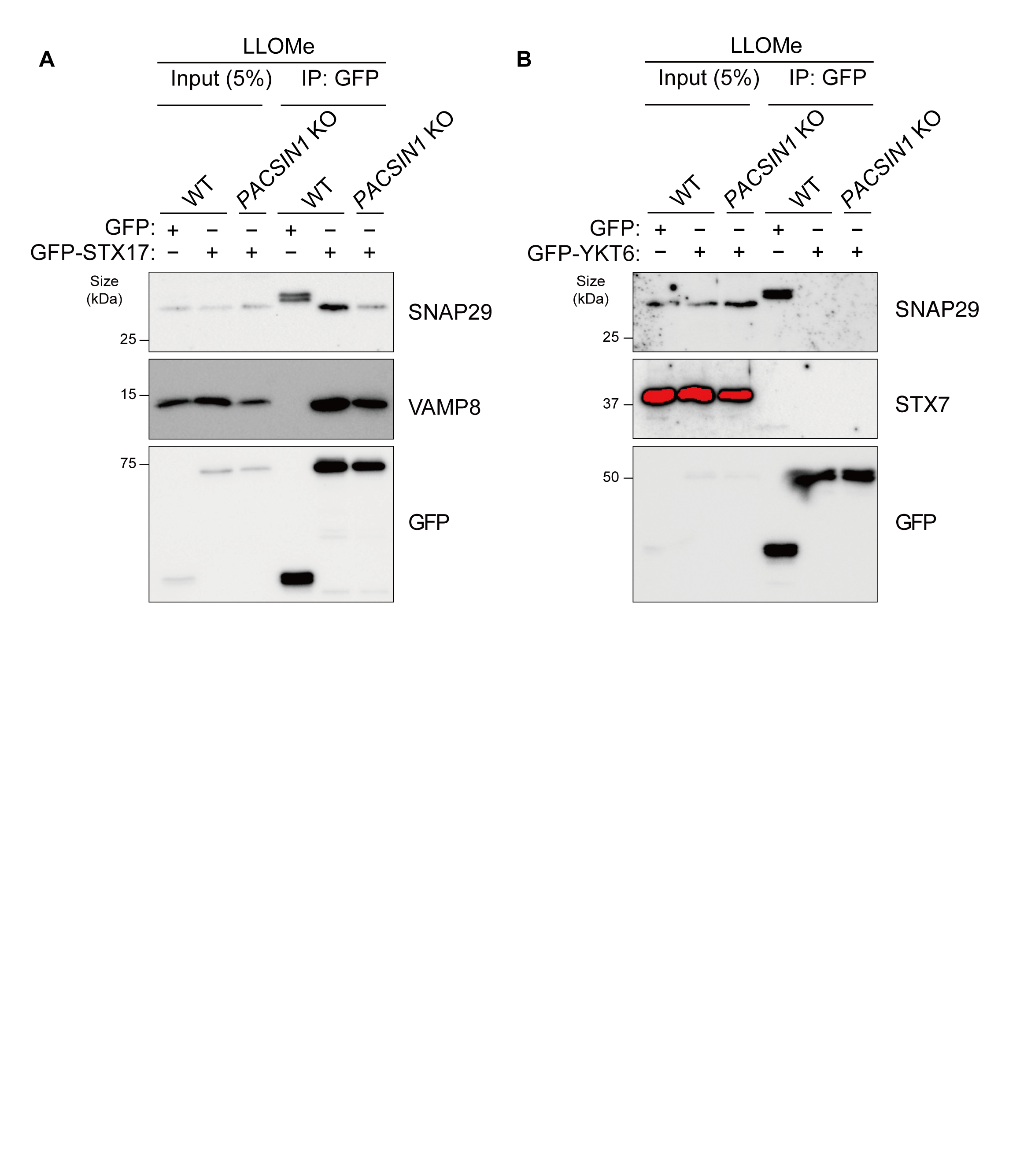

Supplement: S6 Fig — (A) WT or PACSIN1 KO HeLa cells transiently expressing GFP-STX17 were treated with 1 mM LLOMe for 1 h. The lysates were immunoprecipitated with GFP-trap beads and immunoblotted with the indicated antibodies. (B) WT or PACSIN1 KO HeLa cells transiently expressing GFP-YKT6 were treated with 1 mM LLOMe for 1 h. The lysates were immunoprecipitated with GFP-trap beads and immunoblotted with the indicated antibodies. (TIF) [file pgen.1010264.s006.tif]

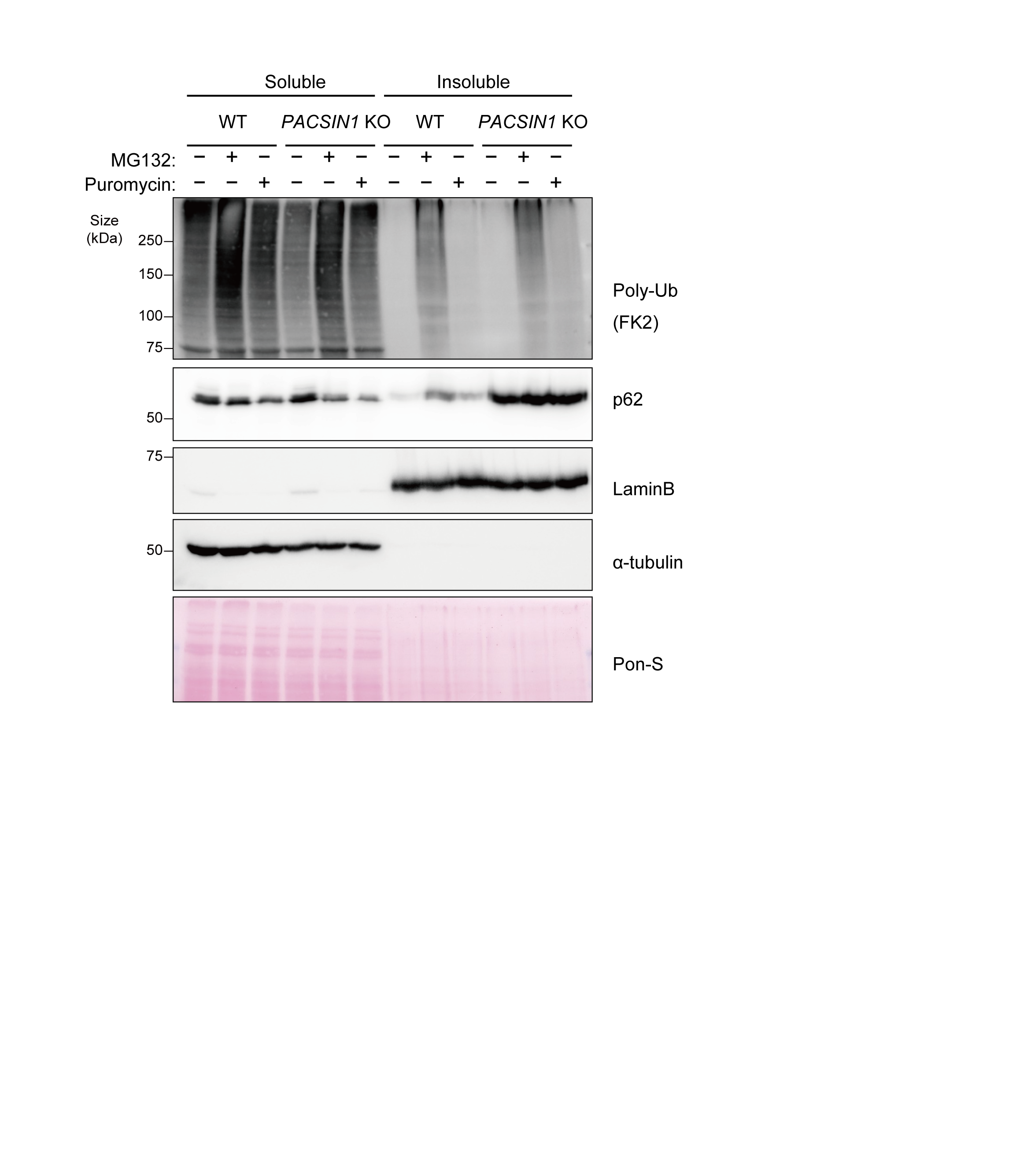

Supplement: S7 Fig — WT and PACSIN1 KO HeLa cells were treated with 20 μM MG132 for 6 h or 2.5 μg/mL puromycin for 6 h. The lysates were separated to detergent-soluble and detergent-insoluble fraction, and then immunoblotted with the indicated antibodies. (TIF) [file pgen.1010264.s007.tif]
