# Supplementary material for: PACSIN1 is indispensable for amphisome-lysosome fusion during basal autophagy and subsets of selective autophagy
Source: PLoS Genet. 2022 Jun 30;18(6):e1010264. doi: 10.1371/journal.pgen.1010264 (PMC9246181; doi:10.1371/journal.pgen.1010264)
Supplement: Attachment — Submitted filename: Oe et al., point by point response _PLOS genetics.pdf [file pgen.1010264.s012.pdf]

Reviewer's Responses to Questions

Comments to the Authors:

Please note here if the review is uploaded as an attachment.

Reviewer #1: Referee #? (Remarks to the Author): PGENETICS-D-21-01574

In this review article, the authors implicate that PACSIN1 plays an important role in the activation of autophagy, particularly in the amphisome-lysosome fusion process. They also claim that PACSIN1 at this time has the task of forming lysophagy by binding to the SNARE complex in autophagy.

Overall, it is thought that they specifically demonstrated the role of PACSN1 specifically for amphisome lysosome fusion based on various experiments. I am satisfied with the amount of trustworthy data presented in this paper. However, some flaws in the paper undermine the study's validity, and these can preclude the publication of this article. But, I can agree to accept this paper if the authors can solve all the problems.

Response

First of all, we thank the reviewer for investing tremendous efforts and times to review our paper. We sincerely appreciate the reviewer's constructive and valuable comments. We are also grateful for the reviewer's comments on our manuscript that "I am satisfied with the amount of trustworthy data presented in this paper" and this is really encouraging for us. All comments raised are quite helpful to improve our manuscripts. we believe that revised experiments conducted so far further manifest our conclusion. The specific point-by-point responses are detailed below.

Major concerns:

1. Studies on PACSIN1 have already been conducted in several other papers on the endocytosis recycle together with the endosome and dynamic trafficking of AMPA receptors (AMPARs). In that study, PACSIN1 controls AMPA receptor trafficking in living hippocampal neurons. Since not many studies on PACSIN1 have been published, it is necessary to consider whether there is a correlation between the researcher's study and the genes already published in the paper mentioned below.

PACSIN1 regulates the dynamics of AMPA receptor trafficking. Widagdo J, Fang H, Jang SE, Anggono V. Sci Rep. 2016 Aug 4;6:31070.

PICK1 interacts with PACSIN to regulate AMPA receptor internalization and cerebellar long-term depression. Anggono V, Koç-Schmitz Y, et al. Proc Natl Acad Sci U S A. 2013 Aug 20;110(34):13976-81.

Native KCC2 interactome reveals PACSIN1 as a critical regulator of synaptic inhibition. Vivek Mahadevan, C Sahara Khademullah, et, al. Elife. 2017 Oct 13;6:e28270.

Response

We thank the reviewer for pointing this out. As the reviewer suggested, we examined a potential correlation between function of PACSIN1 on autophagy and AMPA receptor trafficking. We focused on PICK1 which regulates AMPA receptor trafficking together with PACSIN1, and analyzed autophagy flux in PICK1 KD cells. We also conducted KCC2 knockdown, but the expression of KCC2 in Hela cells is too low to knockdown efficiently. We found that knockdown of PICK1 by using two independent siRNA didn't affect the autophagy flux as shown in revised S2 Fig. These results suggest that a role of PACSIN1 in autophagy can be distinguished from that in AMPA receptor trafficking. We clearly stated this important point in page 6, line 1 as well. Thank you!

2. Fig.2. In the PACSIN1 KO data (Fig. 2 AB), it can be seen that the number of cells significantly decreased compared to the Control. Could it be due to other cell death there? apoptosis. necroptosis, pyroptosis, etc... Also, the cathepsin D activity in PACSIN1 KO seems to be all different (Fig. 2B). Some appear to be weaker than control. If it is PACSIN1 KD, it is partially understandable, but if it is PACSIN1 KO, it can be a little problematic. By staining the nucleus with DAPI, it is necessary to show whether the number of cells and the cell status affected by apoptosis (apoptotic body), etc. Be clear about this part.

Response

We checked the cell survival/proliferation between WT and two independent PACSIN1 KO cell lines using by WST-8 assay. Actually, we could not see any statical differences of cell proliferation/survival between WT and two PACSIN1 KO cell lines from 3 independent experiments as shown in revised S1D Fig., while both PACSIN1 KO cells consistently showed impaired autophagy as shown in revised Fig 1A and B, S1C Fig. Due to this reason, we did not further examine the possible involvement of Pacsin1 in cell death. We believe that this conclusion is reasonable and acceptable for the reviewer. This observation is clearly stated in our revised manuscript in Page 5, line 26. Thank you!

Regarding the concern about cathepsin B activity, we found even WT Hela cells show some variation of Magic Red intensity between cells indicated as arrows and arrowheads in revised Fig.2C and S3A Fig, implying that lysosome activity changes among cells by unknown factors. This point was mentioned in the result section in page 6, line32. Therefore, we think it is reasonable to compare the average intensity of Magic red from proper and large numbers of cells. In our experiment, we quantified Magic red intensity from more than 100 cells and repeated three times. To further confirm that lysosome activity is not affected by PACSIN1 deletion, we further examined additional one cell line and both PACSIN1 KO showed similar Magic red intensity compared with WT (revised Fig.2D and S3B Fig). This result together with our lysotracker results (Fig 2A and 2B) support our conclusion that PACSIN1 depletion does not affect lysosomal activity.

3. Fig.5. If PACSIN1 binds to both SNARE complexes under lysophagy, why does SNAP29 increase although STX7 binding decreases in Figure 3B? What does this mean? No specific mention is made anywhere.

Response

Thank you for the insightful suggestion. Actually, in Figure3B, we examined the assembly of SNARE complex under basal condition but not lysophagy condition. Thus we first examined the assembly of SNARE during lysophagy. Similar to the basal condition, we observed the interaction of STX17 with VAMP8 and SNAP29 is decreased in PACSIN1 KO cells compared to WT during lysophagy as shown in revised S6A Fig. Intriguingly, the interaction of YKT6 with STX7 and SNAP29 is not detected during lysophagy as shown in revised S6B Fig. These results suggest that STX17 complex rather than YKT6 complex is mainly utilized for lysophagy and PACSIN1 is required for the assembly of STX17 complex. We have inserted these results and insights in page 10, line9.

As the reviewer pointed, why interaction between YKT6 and SNAP29 is increased in PACSIN1 KO is currently unknown. We speculate that PACSIN1 has another unknown function through YKT6 and SNAP29. For instance, it has been shown that YKT6 and SNAP29 are required for exsosome or autophagosome secretion (Gross et al., Nat Cell Biol, 2012; Martinez et al., Oncotarget, 2016; kimura et al., EMBO J, 2017). We would like to address the possible involvement of PACSIN1 including exosome secretion in future study. Nevertheless, we would like to emphasize that trans-SNARE assembly between Qa-SNARE and R-SNARE of STX17 and YKT6 complex, which are essential for autophgosome/amphisome fusion with lysosome is commonly compromised in PACSIN1 KO cells. These points and questions are included in the revised manuscript in page 8, line26.

4. Optional request. Most of the research on the lysophagy environment is common to see in detail by giving a stimulus such as LLOMe (Please refer to the references provided). It needs to be clarified, if possible. (This is the biggest part of the researcher's availability, so if time and circumstances are impossible, you do not have to do it.)

Quantitative proteomics reveals the selectivity of ubiquitin-binding autophagy receptors in the turnover of damaged lysosomes by lysophagy. Vinay V Eapen, Sharan Swarup, Melissa J Hoyer, Joao A Paulo, J Wade Harper. Elife. 2021 Sep 29;10:e72328.

The ubiquitin-conjugating enzyme UBE2QL1 coordinates lysophagy in response to endolysosomal damage. Lisa Koerver

Chrisovalantis Papadopoulos, et al. EMBO Reports (2019)20:e48014

ESCRT-mediated lysosome repair precedes lysophagy and promotes cell survival. Maja Radulovic, Kay O Schink, et al. The EMBO Journal (2018)37:e99753

Response

Thank you for the comment. As you mentioned, most of the lysophagy utilizes LLOMe as a drug to specifically induce lysosomal damage. We have inserted the relevant references including ours and others (Maejima et al., EMBO, 2013; Nakamura et al., Nat Cell Biol, 2020; Chauhan et al., Dev Cell, 2016; Eapen et al., Elife, 2021; Koerver et al., EMBO reports, 2019; Radulovic., EMBO J, 2018) in page 9, line28. Moreover, we also briefly described how LLOMe damage lysosomes in page 9, line 30 in the revised manuscript. Thank you!

Minor concerns:

1. No page number. Please insert the page number.

Response

We have now inserted the page number.

2. Fig. 3A. Statistical data is required if there are several electron microscope data.

Response

We thank the reviewer for pointing this out. We counted the number of lysosome and amphisome both in WT and Pacsin1 KO cells from total 80 images of two independent experiments and the quantitative data is now summarized in revised S4A Fig. We found that Pacsin1 KO cells have indeed more amphisomes compared to WT. This result together with the immunofluorescence shown in Fig 3B. corroborates our observation that Pacsin1 KO cells have the accumulated amphisomes. Thank you!

3. Fig. 6. For research on Parkin's related mitophagy (autophagy), it is a reasonable experimental method to give a specific stimulus such as CCCP. It is meaningless to see only PACSIN1 KO in the state of giving nothing. Please make this part clear.

Response

We apologize that we forgot to describe the name of reagent used in revised Fig 6G and 6H. We treated cell with a respiratory chain uncoupler, Valinomycin that has been extensively used to induce mitophagy and we now clearly indicate the name of drug. Thank you!

Reviewer #2: In this manuscript, authors found PACSIN1 regulate the fusion between amphisome and lysosome, thus, control basal autophagic degradation and some but not all selective autophagy. The most surprising and important finding of this study is there are two paths for autophagic flow, one for basal autophagy and one for starvation induced autophagy, and these two paths can be distinguished by PACSIN1 dependency. Overall, this is a conceptually novel study backed by solid evidence, I like to suggest a few experiments to further improve this study.

Response

First of all, we thank the reviewer for investing tremendous efforts and times to review our paper. we sincerely appreciate the reviewer's constructive and valuable comments. We are also grateful for the reviewer's comments on our manuscript that "Overall, this is a conceptually novel study backed by solid evidence" and this is really encouraging for us. All comments raised are quite helpful to improve our manuscripts. we believe that revised experiments conducted so far further manifest our conclusion. The specific point-by-point responses are detailed below.

1. Amphiosome can be distinguished from lysosome by lack some of lysosome marker proteins. The accumulation of amphisome in PACSIN1 KO cells need to be better characterized by staining cells with proteins confined to lysosome compartment.

Response

We thank the reviewer for pointing this out. This is really important point. Actually, we have tested the staining for several other lysosomal proteins including LIMP2, but we could not find good markers confined to lysosomes. Many lysosome markers are partly expressed in late endosomes and this is general technical problem. Therefore, by following the previous amphisome paper, we distinguished amphisomes and lysosome based on their structural differences using electron microscopic data. We counted the number of lysosome and amphisome both in WT and Pacsin1 KO cells from total 80 images of two independent experiments and the quantitative data were now summarized in revised S4A Fig. We found that Pacsin1 KO cells have indeed more amphisomes compared to WT. This result together with the immunofluorescence shown in Fig 3B. corroborates our observation that PACSIN1 KO cells have the accumulated amphisomes. Thank you!

2. Does PACSIN1 also regulate the removal of protein aggregate in mammalian cells?

Response

We thank the reviewer for pointing this out. First, similar to *C. elegans*, we examined whether PACSIN1 is also required for the clearance of an aggregation prone protein, α-synuclein in mammalian cells. For this purpose, we treated cells with α-synuclein fibril labeled with ATTO 550 (PFF 550) and monitored the turnover of the fibril at 0, 24 and 48 hours after treatment.

We found that PACSIN1 KO cells contains significantly large numbers of α-synuclein fibril compared to WT cells as shown in revised Fig 6C and 6D, suggesting that PACSIN1 is required for the removal of α-synuclein aggregates.
We also examined whether PACSIN1 is required for the clearance of other protein aggregates. It is well known that protein aggregates generated by inhibition of the ubiquitin-proteasome system or protein translation is degraded by autophagy (called aggrephagy). We compare the protein aggregate between WT and PACSIN1 KO cells by using aggresome detection kit and WB, respectively, and found that PACSIN1 KO showed increased protein aggregates compared to WT as shown in revised Fig. 6E and 6F, and S7 Fig. All these data suggest that PACSIN1 also regulates the removal of protein aggregate also in mammalian cells through aggrephagy. Thanks to the reviewer's suggestion, since we clearly demonstrate the role of PACSIN1 in aggrephagy, in addition to lysophagy, we changed title to "PACSIN1 is indispensable for amphisome-lysosome fusion during basal autophagy and subsets of selective autophagy". Thank you!

3. Does F-BAR domain required for amphisome/lysosome fusion? Along this line, does F-BAR domain required for localization of PACSIN1 on autophagosome and lysosome?

Response
Thank you for this important comment. Although we tried several times, so far, we failed to obtain the cell line to express proper amount of PACSIN1 lacking F-BAR domain in PACSIN1 KO cells (delta F-BAR) for the functional rescue experiment, probably because delta F-BAR protein is somewhat unstable in this condition. Therefore, we examined the localization of delta F-BAR PACSIN1 by expressing GFP tagged construct in WT cells and found that most delta F-BAR PACSIN1 is ectopically localized in nucleus (revised S5E Fig), suggesting that F-BAR domain is required for proper localization of PACSIN1 and thus presumably essential for its function. On the other hand, since we also found that F-BAR is dispensable for SNAP29 interaction in revised S5F Fig. we could not completely rule out the possibility that small amount of delta F-BAR localize on autophagosomes/amphisomes and lysosomes and function to assemble SNARE complex. These results and possibilities are all included in the revised manuscript in page 11, line 32. Thank you!

4. The figure need to be better labeled.

Response
We have improved the labeling of figures. We separately labeled pictures and graphs. In addition, we also added size markers and the indication to detected proteins in western blot images. Thank you!

Reviewer #3: Major comments

Response

We thank the reviewer for investing tremendous efforts and times to review our paper. we sincerely appreciate the reviewer's constructive and valuable comments. All comments raised are quite helpful to improve our manuscripts. we believe that revised experiments conducted so far further manifest our conclusion. The specific point-by-point responses are detailed below.

It will help understanding if the authors add more details about the quantification of LC3 flux assay and autophagic flux (Fig 1A and B). Given the graphic doesn't show the increase in LC3-II in PACSIN1 KO.

Response

We thank the reviewer for pointing this out and we are sorry for the lack of detailed explanation about the quantification of LC3 flux assay. By following the standard protocol, the autophagic flux was calculated by subtracting the LC3-II value in the absence of BafA1 from that in the presence of BafA1 in basal or starvation medium both in WT and PACSIN1 KO cells. This method is now clearly described in results and the Material and method. For the better understanding the comparison, we also added the lane numbers in Fig.1A and S1C Fig. And basal LC3-II levels were clearly increased in PACSIN1 KO1 and KO2 (Fig.1A lane 5, S1C Fig. lane 5, respectively) compare to WT (lane1). We have added these explanations in the result section. Thank you!

Minor comments.

-Results section (Loss of PACSIN1 impairs autophagic activity): Please add Supplemental Figure 1 after CRISPR-Cas9 system.

Response

Thank you for your suggestion. We have now changed the order of sentence and added supplementary Figure1 (now S1A and S1B Fig) just after mentioning CRISPR/Cas9 system. Thank you!

-PACSIN1 KO: Is it a single clone? or the authors isolate multiple independent clones to confirm phenotypes across the clones avoiding an artifact of a single clone. Please clarify this in the new version.

Response

We thank the reviewer for pointing this out. We have additionally generated one independent PACSIN1 KO (total 2 independent clones) and another clone showed defect in basal autophagy flux as shown in revised Fig 1A and 1B, S1C Fig., suggesting PACSIN1 indeed is required for basal autophagy.

-Fig. 1A: It will help if the authors add the kDa (size) in the western blot for each protein and indicate the LC3-I and II bands.

Response

Thank you for pointing this out. We have now inserted the kDa and indication of LC3-I and II in the western blot.

-Supplemental Fig. 1B: Please correct the size (kDa) of alpha-tubulin to ~50kDa.

Response

We have corrected. Thank you!

-Supplemental Fig. 1C: It will help if the authors add the alpha-tubulin as a loading control.

Response

We are sorry not to include the loading control. Now we have inserted Ponceaus-S as a loading control. We and other laboratory use Ponceaus as a loading control (Yung et al., PLoS ONE, 2011; Fiorini et al., Apotosis, 2013; Chou et al., PNAS, 2011; Fujita et al., JCS, 2021; Nakamura et al., Nat Cell Biol, 2020) and the usage of Ponceaus-S as a loading control is well accepted in cell biological study. So, we hope our explanation and the use of Ponceaus-S are acceptable for the reviewer. Thank you!

-Fig. 1B and D: It will help if the authors add the kDa (size) in the western blot for each protein and probe the membrane with alpha tubulin instead show the Ponceaus-S.

Response

Now we have added the kDa in the western blot for each protein and probe. As we mentioned in the previous section, we and other laboratory use Ponceaus as a loading control (Yung et al., PLoS ONE, 2011; Fiorini et al., Apotosis, 2013; Chou et al., PNAS, 2011; Fujita et al., JCS, 2021; Nakamura et al., Nat Cell Biol, 2020) and the usage of Ponceaus-S as a loading control is well accepted in cell biological study. So, we hope that this is acceptable. Thank you!

-Fig 1C: Did the authors normalize per cell area? If yes, please add the info to the material and methods.

Response

Thank you for pointing this out. We normalized per cell and added the detailed info how to quantify LC3 dots in the material and method.

-Fig 1D: it will help if the authors indicate the LC3-I and II bands in the western blot.

Response

Thank you for pointing this out. We have now inserted the indications to LC3-I and II in the western blot.

-Fig 1E: Why did the authors treat the cell for 8h with BafA1 instead 2h? as they used in Fig1A, B and D. Please clarify this in the new version.

Response

We thank the reviewer for pointing this out. Actually, 2hr treatment of Baf.A1 used in Fig.1A and B is a golden standard for autophagy flux assay. However, for the autophagosome protection assay, we wanted to make sure to accumulate autophagosomes. Therefore, we treated the cells for longer time. We described this reason in the revised manuscript in page 6, line 12.

-Fig 2A/B: How did the authors normalize the lysotracker and magic red intensity? Per cell area? It will help if the authors add this information to the manuscript.

Response

We are so sorry for the lack of detailed information to quantify lysotracker and magic red intensity. We quantified and normalized those intensity per cell (not area). We have now added those method in the material and methods. Thank you!

-Fig 3A: Please add PACSIN1KO ST into the figure.

Response

We have now added the representative picture of PACSIN1 KO in starvation condition in revised Fig.3A. Thank you!

-Fig 3B: The authors should keep constant the treatment with BafA1. In Fig 1A, B and D they used 2h of treatment, in Fig. 1E 8h and here in Fig. 3B 6h. It will help if the authors clarify why they used different times of BafA1 treatment.

Response

As mentioned above, we normally treated cells with BafA1 for 2hrs to analyze autophagy flux. But for the autophagosome protection assay and electron microscopic analysis, we wanted to make sure to accumulate autophagosome/amphisomes. Therefore, we treated cells with BafA1 for longer period. We described those reasons in the revised manuscript, page 7, line 14.

-Supplemental Fig. 2A: Please add in the legend the cell line used in the experiment.

Response

We have now added the cell line used in the experiment in the figure legend (now S4B Fig). Thank you!

-Supplemental Fig. 2B. Is it possible to do LAMP1/Cd63 and LC3 staining together? It will improve the conclusion.

Response

We thank the reviewer for pointing this out. Indeed, we actually stained LAMP1/CD63 and LC3 together and quantified the colocalization ratio by using CQ1(YOKOKAWA). We added the detailed staining procedure in the material and method. One the other hand, it is well known that late endosome marker, CD63 and lysosome marker LAMP1 are not exclusively expressed in each compartment and have some overlap. Therefore, we also quantified amphisome and lysosome based on the structural differences from pictures of electron microscopy. We counted the number of lysosome and amphisome both in WT and PACSIN1 KO cells from total 80 images of two independent experiments and the quantitative data were now summarized in S4A Fig. We found that PACSIN1 KO cells have indeed more amphisomes compared to WT. This result together with the immunofluorescence shown in Fig 3B. corroborates our observation that PACSIN1 KO cells have the accumulated amphisomes. Thank you!

-Fig 5A: It will improve the result if the authors add a control such as cells treat with BafA1.

Response

We thank the reviewer for pointing this out. Actually, the previous several reports studying YKT6 complex and STX17 complex during autophagosome-lysosome fusion (Itakura et al.,

Cell, 2012; Matsui et al., JCB, 2018; Wang et al., Mol Cell, 2016) did not use Baf.A1 treatment as a control and, as far as we examined, we could not find any other good negative control when we examine the SNARE assembly. Instead, to further validate PACSIN1 is indeed involved in SNARE assembly, we checked SNARE complex during lysophagy in which PACSIN1 function is required. Although we found YKT6 complex themselves are not utilized during lysophagy, PACSIN1 KO cells clearly showed reduced assembly of STX17 complex as shown in S6A and 6B Fig suggesting that similar to basal autophagy PACSIN1 is required for the assembly of STX17 complex. These results are included in page 10, line 8.
