# Supplementary material for: PACSIN1 is indispensable for amphisome-lysosome fusion during basal autophagy and subsets of selective autophagy
Source: PLoS Genet. 2022 Jun 30;18(6):e1010264. doi: 10.1371/journal.pgen.1010264 (PMC9246181; doi:10.1371/journal.pgen.1010264)
Supplement: Attachment — Submitted filename: Referee (05-05-2022)-PGENETICS-D-21-01574.doc [file pgen.1010264.s013.doc]

*Referee #1 (Remarks to the Author):* ***PGENETICS-D-21-01574R1***

***Title:*** *PACSIN1 is indispensable for amphisome-lysosome fusion during basal autophagy and subsets of selective autophagy*

**Recommendation:** *I recommend accepting the publication of the thesis as I am satisfied with the authors' sincere responses and specific additional experiments. While doing the review, it was a good time for me too.*
